# Longitudinal co-activation pattern analysis of menstrual cycle-related brain dynamics in primary dysmenorrhea

**DOI:** 10.1016/j.isci.2026.116525

**Published:** 2026-06-26

**Authors:** Huiping Liu, Xing Su, Yanran Chen, Huiyan Gan, Meiling Shang, Xiaotong Chi, Youjun Li, Tao Lu, Ming Zhang, Wanghuan Dun, Zi-Gang Huang

**Affiliations:** 1 Department of Medical Imaging, the First Affiliated Hospital of Xi’an Jiaotong University, Xi’an, Shaanxi 710061, China; 2 School of Future Technology, Xi’an Jiaotong University, Xi’an, Shaanxi 710049, China; 3 The Key Laboratory of Biomedical Information Engineering of Ministry of Education, Institute of Health and Rehabilitation Science, School of Life Science and Technology, Xi’an Jiaotong University, The Key Laboratory of Neuro-informatics and Rehabilitation Engineering of Ministry of Civil Affairs, Xi’an, Shaanxi 710049, China; 4 Research Center for Brain-inspired Intelligence, Xi’an Jiaotong University, Xi’an, Shaanxi 710049, China; 5 Xi’an Jiaotong University Health Science Center, Xi’an, Shaanxi 710049, China; 6 Rehabilitation Medicine Department, The First Affiliated Hospital of Xi’an Jiaotong University, Xi’an, Shaanxi 710061, China

**Keywords:** Neuroscience, Physiology

## Abstract

Primary dysmenorrhea (PDM) is a chronic pelvic pain condition characterized by recurrent painful phases. While abnormal prostaglandin activity is central to its pain mechanism, and previous studies link PDM to central nervous system alterations associated with prostaglandin F2αlevels and pain intensity, the dynamic evolution of brain networks across the menstrual cycle remains unknown. This study employed the co-activation pattern analysis to investigate dynamic brain network characteristics across the menstrual, periovulatory, and luteal phases. Correlation analyses were performed between CAP metrics, pain scores, and PGF2α levels. Our results revealed that dynamic alterations of brain networks in patients with PDM exhibited trending changes throughout the menstrual cycle. Notably, the default mode network, salience network, sensorimotor network, and central executive network demonstrated significant periodic changes, which correlated with fluctuations in pain and PGF2α levels. This longitudinal study elucidates the dynamic neural mechanisms of patients with PDM, offering insights for early intervention strategies.

## Highlights

• Patients with PDM exhibited trending brain network changes across the menstrual cycle
• The DMN, SN, SMN, and CEN play important roles in the neural mechanisms in PDM
• Dynamic brain alterations are associated with pain intensity and PGF₂α levels

## Graphical abstract

**Figure:**
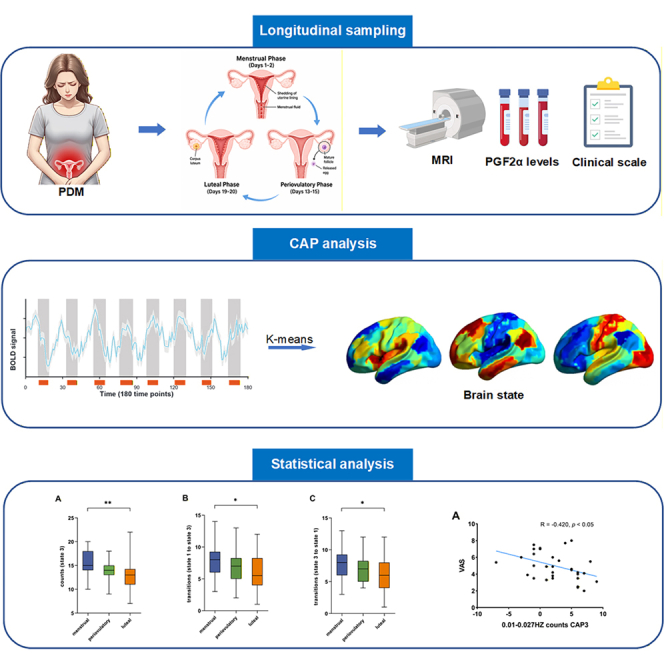

## Introduction

Primary dysmenorrhea (PDM) affects 50–90% of women worldwide, with half experiencing moderate-to-severe pain.^1^ PDM negatively impacts patients’ quality of life, work efficiency, and psychological conditions.^2^^,^^3^ The mechanism of PDM is complex, involving the central nervous system, prostaglandins (PGs), metabolism, and other factors.^2^^,^^4^^,^^5^ Prolonged nociceptive input to the central nervous system can induce functional and structural alterations, contributing to central sensitization.^6^^,^^7^ The menstrual cycle consists of the menstrual, follicular, ovulatory, and luteal phases. PDM encompasses recurrent spontaneous painful (“on”) and pain-free (“off”) states and is thus a good clinical model to study state- and trait-related changes of pain in the brain.

Widespread alterations in structural and functional topology are observed across the whole brain in patients with chronic pain, leading to the large-scale reorganization of brain networks.^8^^,^^9^

Accordingly, brain network approaches have great potential for providing system-level insights into brain dynamics.^10^ In patients with PDM, previous studies have demonstrated aberrant functional brain network organization, primarily involving the default mode network (DMN), central executive network (CEN), and salience network (SN).^11^^,^^12^^,^^13^^,^^14^ In addition, menstrual cycle-dependent structural and functional brain abnormalities have been documented, including alterations in gray matter volume (GMV) and reward system activity.^7^^,^^15^^,^^16^^,^^17^ However, most existing studies have relied on cross-sectional designs and static functional connectivity (FC) analyses. Such approaches neglect the temporal dynamics of brain function and related clinical and biochemical measures, including pain severity, prostaglandin levels, and emotional states.^18^^,^^19^ Longitudinal studies are essential to elucidate dynamic brain changes across the menstrual cycle, thereby advancing understanding of its neural mechanisms and identifying optimal therapeutic time windows. To more sensitively capture dynamic brain network alterations across the menstrual cycle in patients with PDM, the present study incorporated brain imaging and clinical data collected across multiple menstrual phases. We applied a co-activation pattern (CAP) analysis to characterize dynamic brain network features and further investigated their associations with pain severity and prostaglandin levels.

Conventional static FC analysis assumes that the relationships between neural regions remain consistent over time, overlooking the influence of temporal fluctuations on pain.^20^ Dynamic analysis may offer a more sensitive and nuanced approach to characterizing brain network dynamics in PDM by sensitively capturing brain alterations and providing a more comprehensive examination of brain network activity.^21^^,^^22^ CAP analysis, a data-driven method, extracts CAPs of brain activity through cluster analysis, overcoming the predefined time-window limitation of traditional sliding-window correlation analysis.^23^^,^^24^ CAP considers the non-stationarity of brain activity and can capture dynamic changes in brain networks.^23^^,^^24^^,^^25^ Using CAP analysis, our previous study identified abnormal brain network interactions in patients with PDM during the pain-free phase compared with healthy controls (HCs).^26^ However, most neuroimaging studies of PDM have relied on cross-sectional designs, limiting insights into disease progression. Therefore, the present study further investigated the brain CAPs of patients with PDM across three different menstrual-cycle phases, aiming to characterize menstrual cycle-related dynamic alterations in brain activity and to provide insights that may inform the early prevention and intervention of menstrual symptoms. Additionally, resting-state fMRI signals contain multiple frequency components reflecting distinct neural processes.^27^ Previous rs-fMRI studies have examined brain activity in the 0.01–0.08 Hz frequency band.^28^^,^^29^ The slow-4 (0.027–0.073 Hz) and slow-5 (0.01–0.027 Hz) frequency bands are predominant in gray matter and are particularly sensitive to disease-related alterations in brain function.^27^^,^^30^^,^^31^ Therefore, this study focused on both the typical frequency band (0.01–0.08 Hz) and its subbands, slow-4 and slow-5, to explore brain network changes in PDM.

Our study aims to identify a dynamic brain network CAP in patients with PDM across menstrual phases and integrate peripheral biomarkers with central neural activity. We hypothesize that: (1) dynamic brain network activation varies with the menstrual cycle in patients with PDM, (2) the alterations in brain networks across the menstrual cycle are associated with pain perception and PGFα levels in patients with PDM.

## Results

### Demographic and clinical characteristics of participants

A total of 46 participants completed MRI scans at three time points. Participants exhibiting head movements greater than 2 mm or 2° during any scan were excluded. As a result, 12 participants were excluded due to excessive head motion, and the final fMRI analysis included 34 participants. The demographic and clinical characteristics of patients with PDM are presented in Table 1. Participants with incomplete scale or blood sample data at any of the three time points were excluded from the analyses of scale scores and PGF2α levels. A total of 30 participants were included in the analyses of scale scores and PGF2α levels. Notably, patients with PDM experience pain during the menstrual phase, while pain scores are zero during the periovulatory and luteal phases. Statistical analysis revealed differences in SAS scores (*F*_2,58_ = 9.673; *p* < 0.001; η_p_^2^ = 0.250) and SDS scores ((*F*_2,58_ = 12.909; *p* < 0.001; η_p_^2^ = 0.308) across the three phases. The SAS score during the menstrual phase was significantly higher than that during the periovulatory phase (*p* = 0.004), and the SAS score during the luteal phase was also significantly higher than that during the periovulatory phase (*p* = 0.002). Similarly, the SDS score during the menstrual phase was significantly higher than that during the periovulatory phase (*p* = 0.001), and the SDS score during the luteal phase was significantly higher than that during the periovulatory phase (*p* = 0.001). One-way ANOVA revealed no significant difference in PGF2α levels.

**Table 1:** Demographic and clinical characteristics of participants

Outcome	Mean (SD)			P-value					
	Menstrual	Periovulatory	Luteal	F	partial η^2^	ANOVA *p*	Menstrual vs. Periovulatory	Menstrual vs. Luteal	Periovulatory vs. Luteal
Age (y)	24.12 ± 2.06	/	/	/	/	/	/	/	/
BMI (kg/m^2^)	20.01 ± 1.88	/	/	/	/	/	/	/	/
Age at menarche (y)	13.24 ± 1.46	/	/	/	/	/	/	/	/
Menstrual cycle (d)	29.42 ± 1.68	/	/	/	/	/	/	/	/
Duration of menstrual pain (h)	21.18 ± 17.64	/	/	/	/	/	/	/	/
VAS	4.95 ± 1.65	0.00 ± 0.00	0.00 ± 0.00	/	/	/	**< 0.001**	**< 0.001**	/
SAS	35.22 ± 8.95	28.43 ± 5.62	33.19 ± 6.45	9.673	0.250	**< 0.001**	**0.004**	0.532	**0.002**
SDS	37.91 ± 8.91	30.38 ± 5.50	35.81 ± 7.19	12.909	0.308	**< 0.001**	**0.001**	0.413	**0.001**
PGF 2α	14.40 ± 11.26	13.67 ± 9.81	13.06 ± 9.64	0.817	0.027	0.447	1.000	0.753	1.000

The data shown are the mean ± SD. Statistical significance was set at *p* < 0.05 using the one-way repeated measures ANOVA. The results of the pairwise comparisons were Bonferroni-corrected. During the collection of scale data, the extended duration of the study resulted in occasional oversights, leading to incomplete recording or loss of some data. Consequently, complete datasets including both scale assessments and blood samples were available for 30 participants. VAS visual analog scale, PGF 2α prostaglandin F2α, SAS Self-Rating Anxiety Scale, SDS Self-Rating Depression Scale.

### Cluster analysis yielded three recurring CAP states at different frequency bands

**Figure 1:**
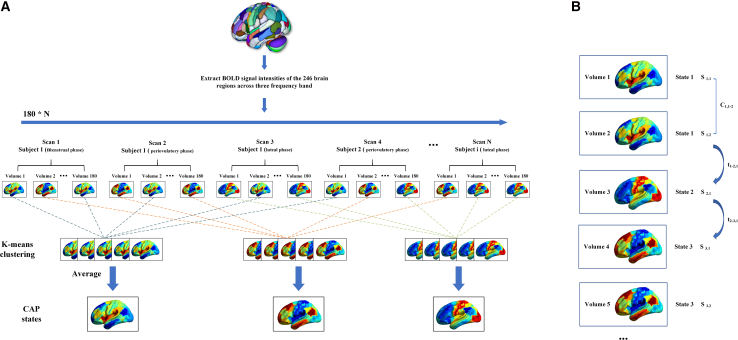
Schematic illustration of the CAP analysis pipeline and the calculation of dynamic CAP metrics (A) CAPs analysis pipeline. The application of k-means clustering to the concatenated time series of the three phases to identify recurring brain states across time points with similar whole-brain coactivation patterns for each frequency band. Volume refers to a single 3D brain scan obtained at a specific time point during the fMRI acquisition. The average volumes were computed within each cluster, resulting in a CAP state. The 180 represents the number of time points, and N represents the number of MRI scans of all scans. (B) The dynamic properties of CAP states. s, state; c, counts; t, transition. S_A,j_ represents that CAP state A appeared j times, e.g., S_1,2_ indicates that state 1 appeared 2 times. C_A,m-n_ represents the duration of the n TRs (time ranges) during the m-th occurrence of CAP state A, e.g., C_1,1-2_ indicates that the first occurrence of state1 lasted for two TRs. Different time points TR correspond to different brain states. And t_A-B,h_ represents that the transition from CAP state A to CAP state B occurred h times, e.g., t_1-2,1_ indicates that the transition from CAP state 1 to CAP state 2 occurred once. Fraction of state A = maximum j/ total volumes. Persistence of state A = (∑n)/maximum m. Counts of state A = maximum m. Transitions of state A = maximum h.

**Figure 2:**
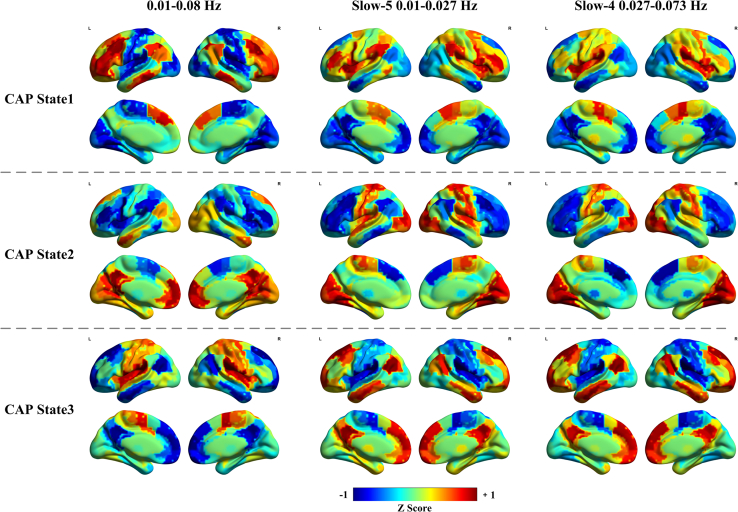
The frequency-specific effects between the 0.01–0.08 Hz frequency band and two frequency sub-bands (slow-5 and slow-4) within all MRI scans of the patients The three columns show the CAP topographies characteristics of the 0.01–0.08 Hz frequency band, slow-5 and slow-4. CAP, co-activation pattern.

CAP analysis was performed using k-means clustering to identify recurring brain states (Figure 1). The optimal value of k = 3 was determined by evaluating the silhouette scores.^32^ Three recurring CAP states were identified at the 0.01–0.08 Hz frequency band and their two subbands (Figure 2). The order of CAP states in the three frequency bands is defined based on the fraction of time. The first column shows the three CAP topographies within a 0.01–0.08 Hz frequency band. State 1 involves the activation of the CEN and deactivation of the SMN. In state 2, the coactivation of the DMN and the deactivation of the CEN and SN were observed. In state 3, the coactivation of the SN and SMN, and the deactivation of the DMN were observed. The second column shows the three CAP topographies in the slow-5 frequency band. State 1 mainly involves the coactivation of the SN and CEN and the deactivation of the DMN and visual network (VN). In state 2, the coactivation of the SMN and VN and the deactivation of the CEN were observed. In state 3, the activation of the DMN and deactivation of the SMN and SN were observed. The third column shows the three CAP topographies in the slow-4 frequency band. In state 1, the SN and SMN were primarily activated, and the DMN was deactivated. State 2 mainly involves the activation of the VN and deactivation of the DMN and CEN. State 3 involves the activation of the DMN and CEN, and the deactivation of the SMN and SN.

**Figure 3:**
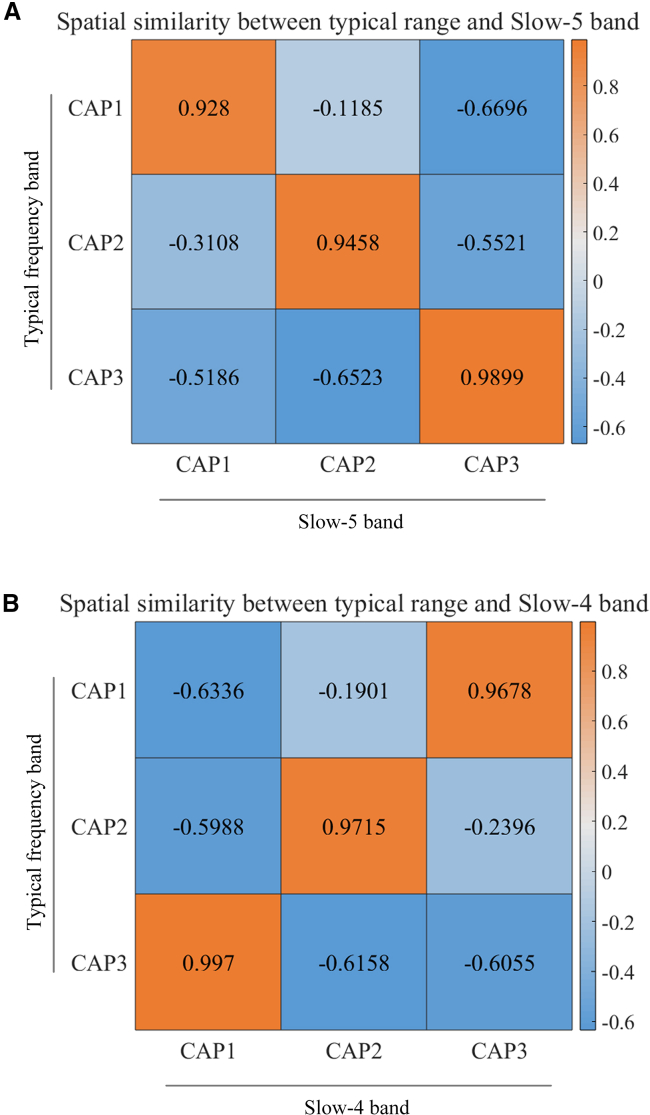
Spatial similarity between 0.01 and 0.08 Hz frequency band and sub-bands (A) Spatial similarity between the slow-5 frequency band and 0.01–0.08 Hz frequency band. In the correlation matrix, the diagonal correlation coefficient is the highest, indicating that CAPs with high spatial similarity between the 0.01–0.08 Hz frequency band (y axis) and the slow-5 band (x axis) maintained a consistent order in terms of the fraction of time. (B) Spatial similarity between the slow-4 frequency band and 0.01–0.08 Hz frequency band. Between the 0.01–0.08 Hz frequency band (y axis) and the slow-4 bands (x axis), the order of the fraction of time with high spatial similarity changed.

We analyzed the spatial similarity of CAP states across different frequency bands to identify the order of fraction of time changes for CAP states across different frequency bands. The spatial similarity of the CAP states between the 0.01–0.08 Hz frequency band and its two sub-bands is presented in Figure 3. We observed that the CAP state with high spatial similarity between the 0.01–0.08 Hz frequency band and the slow-5 band maintained a consistent order in terms of the fraction of time. The diagonal correlation coefficient of the correlation matrix was the highest (Figure 3A). However, the order of the fraction of time with high spatial similarity changed between the 0.01–0.08 Hz frequency band and the slow-4 band. In this case, the highest correlation coefficients were not distributed along the diagonal of the correlation matrix (Figure 3B).

### Characterization of brain dynamic metrics

**Figure 4:**
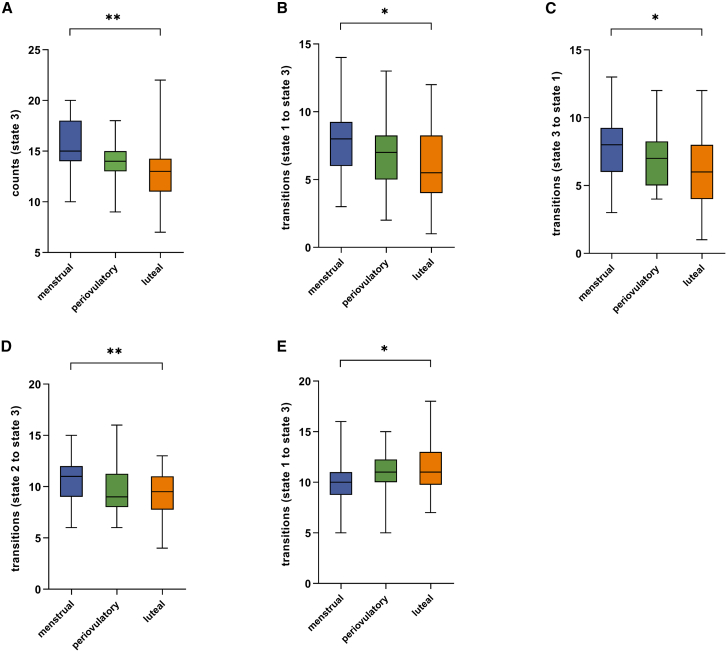
Dynamic metrics differences across three menstrual phases (A–C) Dynamic metrics show significant phase-related differences in the slow-5 band. (D and E) Dynamic metrics show significant phase-related differences in the slow-4 band. Data are presented as box-and-whisker plots. The center line represents the median, box limits indicate the interquartile range, and whiskers indicate the minimum and maximum values. One-way repeated measures ANOVA was used to compare the CAP metrics of patients with PDM at the three time points. In case of significant effects, post hoc analyses were conducted with FDR correction. ∗*p* < 0.05 and ∗∗*p* < 0.01.

The brain dynamic metrics that exhibited statistically significant differences across the three phases are presented in Figure 4. In slow-5 frequency band, statistically significant phase-related differences were observed in the counts of CAP state 3 (*F*_2,66_ = 6.72; *p* = 0.002; η_p_^2^ = 0.17) (Figure 4A), as well as in the transitions from CAP state 1 to state 3 (*F*_2,66_ = 4.08; *p* = 0.021; η_p_^2^ = 0.11) (Figure 4B) and from CAP state 3 to state 1 (Figure 4C) (*F*_2,66_ = 4.43; *p* = 0.016; η_p_^2^ = 0.12). Post hoc analyses further revealed significant differences between the luteal and menstrual phases in the counts of CAP state 3 (*p* = 0.003), transitions from CAP state 1 to state 3 (*p* = 0.027), and from CAP state 3 to state 1 (*p* = 0.035). In the slow-4 frequency band, the analysis revealed statistically significant differences across phases as determined by one-way repeated measures ANOVA for transition from CAP state 2 to state 3 (*F*_2,66_ = 4.62; *p* = 0.013; η_p_^2^ = 0.12) (Figure 4D) and CAP state 1 to state 3 (Figure 4E) (*F*_2,66_ = 3.85; *p* = 0.026; η_p_^2^ = 0.10). Post hoc analyses with Bonferroni adjustment indicated significant differences between the luteal and menstrual phases for the transition from CAP state 2 to state 3 (*p* = 0.007) and CAP state 1 to state 3 (*p* = 0.033).

### Relationship between CAP dynamics and clinical symptoms

**Figure 5:**
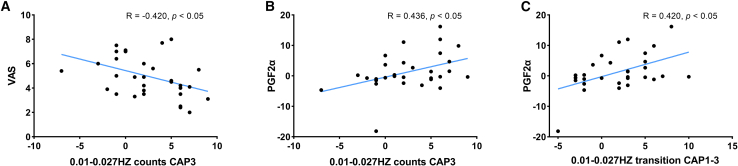
Correlations between dynamic metrics of the slow-5 band and clinical indicators (A) The change in VAS scores between the menstrual and luteal phases was significantly correlated with the change in the CAP state 3 counts between these phases. (B) The change in PGF 2α levels between the menstrual and luteal phases was significantly correlated with the change in the CAP state 3 counts between these phases. (C) The change in PGF 2α levels between the menstrual and luteal phases was significantly correlated with the change in the transitions from CAP state 1 to state 3 between these phases.

A correlation analysis was conducted between the differences in CAP metrics that exhibited phase-specific variations (luteal vs. menstrual phases) and the corresponding differences in clinical indicators across these two phases. The correlation analysis results for the slow-5 band are shown in Figure 5. The counts of CAP state 3 in the slow-5 frequency band were negative correlated with the VAS scores (r = −0.420, *p* < 0.05) (Figure 5A) and positively correlated with the PGF 2α levels (r = 0.436, *p* < 0.05) (Figure 5B). The transitions from CAP state1 to state 3 were positively correlated with the PGF 2α levels (r = 0.420, *p* < 0.05) (Figure 5C). No significant correlations were observed in the typical frequency band or slow-4 band.

## Discussion

This study initially examined the trends in brain co-activation characteristics in patients with PDM across different menstrual phases by applying the CAP analysis method. Results revealed that brain CAPs in patients with PDM change across the menstrual cycle, and these alterations were observed in the slow-4 and slow-5 frequency bands. These cyclic brain dynamic alterations in the slow-5 frequency band were found to be associated with pain intensity and PGF_2_α levels. The longitudinal design provides a more comprehensive exploration of how brain networks dynamically change over time during the menstrual cycle. Our findings may offer insights into brain network dysfunction and neuropathological mechanisms of PDM.

In the slow-5 frequency band, we observed statistically significant phase-related differences in transitions from CAP state 1 to state 3 and state 3 to state 1. Specifically, more transitions occurred during the menstrual phase compared to the luteal phase. CAP state 1 primarily involves the coactivation of the SN and the CEN, while state 3 is characterized by the activation of the DMN. In our previous study, we investigated abnormal dynamic brain network interactions during the pain-free periovulatory phase in patients with PDM compared to HCs using a cross-sectional design.^26^ The previous findings revealed that patients with PDM exhibited significantly more transitions between a CAP state characterized by the activation of the SN and basal ganglia, and another state involving coactivation of the DMN and CEN. In the current longitudinal study, we observed a lower number of transitions from CAP state 1 to state 3 in the slow-4 frequency band during the menstrual phase relative to the luteal phase. In the slow-4 frequency band, the SN and SMN were activated in state 1, the DMN and the CEN were activated in state 3. Normally, activity in the DMN is anticorrelated with activity of the SN, and not correlated with the SMN.^33^^,^^34^ The abnormal increased binding between SN and DMN in pain was suggested in previous studies.^35^^,^^36^^,^^37^ Disrupted DMN-SN FC has been reported in several chronic pain disorders.^38^^,^^39^ Furthermore, cross-network connectivity between the DMN and SMN is positively correlated with clinical pain score.^40^ The SN has been implicated in the detection and integration of emotional and sensory stimuli,^41^ as well as responsible for switching between the DMN and the CEN.^42^^,^^43^^,^^44^ Our results are consistent with previous findings, indicating that the DMN, SN, and SMN are key brain networks involved in the central nervous mechanisms of PDM. It seems that prolonged pain exposure appears to affect brain function, and the alleviation of pain perception is associated with changes in brain network connectivity.^38^^,^^45^^,^^46^^,^^47^^,^^48^ We speculate that during the menstrual phase, the increased transitions between CAP state 1 (in which the SN and CEN are activated) and state 3 (in which the DMN was activated) in slow-5 may disrupt the maintenance of the normal brain state. However, the lower transitions from CAP state 1 (where the SN and SMN are activated) to state 3 (where the DMN and CEN are activated) in slow-4 may underlie the maintenance of abnormal brain states in patients with PDM. Additionally, it is suggested that gray matter-related oscillations primarily occurred in slow-4 and slow-5 bands,^27^ and previous studies have shown that slow-4 and slow-5 bands had different levels of sensitivity to different brain disorders.^30^^,^^49^^,^^50^ Previous studies reported that slow-5 abnormalities are mainly restricted to the ascending pain pathway (thalamus and primary somatosensory cortex), whereas slow-4 alterations involve the DMN and SN.^51^ Nociceptive input may arrive in the cerebral cortex, involving key regions of the SMN and SN network, through the thalamus and parallel pathways. In pain patients, these pathways link the thalamus to the SMN and SN.^52^ In our study, patients with PDM showed abnormal interactions among the CEN, SN, and DMN in the slow-5 band, and between the DMN and SN in the slow-4 band. These alterations may reflect disruptions in ascending pain pathways and suggest that slow-5 and slow-4 bands are particularly sensitive to brain network abnormalities in PDM.

Statistically significant phase-related differences were also observed in counts in the slow-5 frequency band. Specifically, increased counts in CAP state 3 were found during the menstrual phase, in which the DMN was primarily activated. Neuroimaging studies indicate that patients with PDM exhibit persistent alterations in DMN function, which are linked to sensory and affective processing of menstrual pain and may contribute to central sensitization.^53^ We speculate that increased activation of the DMN during the menstrual phase in patients with PDM may be influenced by negative menstrual-related stimuli and contribute to the perception of increased pain intensity.^12^^,^^54^^,^^55^

In the slow-4 frequency band, patients with PDM exhibited significantly more transitions from CAP state 2 to state 3 during the menstrual phase. CAP state 2 was characterized by the activation of the VN, while CAP state 3 involved coactivation of the DMN and CEN. Previous studies have shown that alterations in the VN FC in chronic low back pain may reflect an adaptation/self-adjustment mechanism and cross-model interaction between the visual, somatosensory, motor, attention, and salient networks.^56^ The FC of the left and right CEN shows both overlapping and distinct associations with menstrual pain and menstrual pain interference.^13^ We speculate that the interaction between the DMN, CEN, and VN may be a neural response to the experience of PDM.^57^^,^^58^

Building on our previous research and the results of the current study, we observed that patients with PDM exhibit abnormal brain co-activation not only during the pain-free period^26^ but also show altered brain network interactions when experiencing menstrual pain, relative to the pain-free period. Additionally, as shown in the Figure 4, the co-activation indices demonstrate either an increasing or decreasing trend across the three menstrual phases, suggesting that the changes in brain CAPs may be a long-term and dynamic process.

We found the changes of PGF2α levels were associated with alterations of the counts in CAP state 3 in the slow-5 frequency band. The PGF2α levels were also associated with the transitions from CAP state 1 to state 3. Although PGF2α levels did not show significant differences across the three menstrual phases in patients with PDM, a slight elevation was still observed during the menstrual phase. The levels of menstrual fluid and PGs fluctuate greatly throughout the menstrual cycle, and the time of sample collection may also affect the results.^19^ The overproduction of uterine PGs leads to myometrial hypercontractility, resulting in ischemia and hypoxia of the uterine muscle, and, ultimately pain.^59^ Pain stimulation may be one of the factors contributing to alterations in brain network characteristics in patients with PDM.^60^ Additionally, inflammation is a major factor of abdominal pain and PGs are among the key molecular mediators involved in the mechanisms of visceral pain.^61^ Previous studies have demonstrated that PGs have a plethora of actions in central nervous system cells, differently influencing the progression of inflammation and neuronal death or survival.^62^ Among them, PGF2α can serve as a pro-inflammatory mediator due to its high expression in inflamed tissues and ability to induce inflammatory symptoms.^63^ Consistent with this, the effectiveness of nonsteroidal anti-inflammatory drugs in relieving the pain of PDM by inhibiting prostaglandin production.^19^ PGF2α mediates its biological effects, including pain sensitization, by binding to the FP receptor, a Gq-protein-coupled receptor.^64^^,^^65^ Activation of this receptor triggers Gq-protein pathways, resulting in increased intracellular signaling and potential amplifying nociceptive transmission.^66^^,^^67^ PGF2α can be formed *in vivo* from arachidonic acid through both isoforms of COXs, namely cyclooxygenase-1 (COX-1) and cyclooxygenase-2 (COX-2), COX-2-mediated prostaglandin synthesis contributes to elevated PGF2α levels in dysmenorrhea.^68^^,^^69^ In addition, COX-2 is upregulated during inflammatory responses and plays an important role in neuroinflammation.^70^^,^^71^ The association between serum PGF2α levels and CAP metrics may reflect the coupling between peripheral inflammatory processes and central neural plasticity involved in pain modulation. Moreover, a negative correlation was found between VAS scores and the counts of CAP state 3 in the slow-5 frequency band. The DMN was activated in the CAP state 3. As a major component of the dynamic pain connectome,^72^ the DMN shows decreased connectivity during chronic pain, suggesting a maladaptive and persistent state of heightened attention or vigilance.^39^^,^^73^ Our findings suggest that variations in pain intensity between the pain and pain-free phases in patients with PDM are associated with functional alterations in the DMN. The increased counts of CAP state 3 during the menstrual phase may result from pain stimulation and central sensitization.^53^^,^^74^

In conclusion, this study demonstrates the dynamic changes in the CAPs of patients with PDM across the menstrual cycle. The dynamic interactions of brain networks may be affected by the menstrual cycle, exhibiting an obvious trend. Pain-related brain networks, including the DMN, SN, SMN, and CEN, play a crucial role in these periodic alterations. The cyclical changes in the coactivation of brain networks may be influenced by fluctuations in PGF2α levels and pain stimuli. Investigating the characteristics of dynamic network interactions across different menstrual phases enhances our understanding of the central nervous mechanisms underlying PDM and may inform the development of targeted interventions.

### Limitations of the study

We note several limitations of our study. First, our study population was mostly recruited from the researcher’s hospital or a nearby university, which resulted in a homogeneous study population and limited the generalizability of the results. Second, this study focused on changes in brain network characteristics of patients with PDM across three menstrual phases. Future studies should incorporate data from HCs across the same menstrual phases to compare the trends of changes between groups and examine potential differences in the predominantly activated brain networks. Such comparisons may offer more comprehensive insights into the central nervous mechanisms of PDM.

## Resource availability

### Lead contact

Further information and requests for resources and reagents should be directed to and will be fulfilled by the Lead Contact, Zi-Gang Huang (huangzg@xjtu.edu.cn).

### Materials availability

This study did not generate new unique reagents or materials.

### Data and code availability

#### Data

All data reported in this paper are available from the lead contact upon request.

#### Code

All original code has been deposited at Zenodo and is publicly available as of the date of publication. DOIs are listed in the key resources table.

#### Additional information

Any additional information required to reanalyze the data reported in this paper is available from the lead contact upon request.

## Acknowledgments

This work was supported by the STI 2030-Major Projects (nos. 2022ZD0208500 and 2021ZD0201300), the NSF of China (nos. 62401456 and 12305051), the Shaanxi Fundamental Science Research Project for Mathematics and Physics (grant no. 22JSQ037), the Institutional Foundation of The First Affiliated Hospital of Xi’an Jiaotong University (2024-MS-25), the Clinical Research Project Funds for the First Affiliated Hospital of Xi’an Jiaotong University (XJTU1AF-CRF-2022-023), and the Health Research and Innovation Capacity Strengthening Platform Program of Shaanxi Province (no. 2023PT-09).

## Author contributions

H.L.: data curation, formal analysis, writing – original draft. X.S.: formal analysis, and writing – original draft. Y.C.: data curation and visualization. H.G.: validation and data curation. M.S.: writing – review and editing and study conception. X.C.: writing – review and editing and data analysis. Y.L.: methodology. T.L.: validation. M.Z.: project administration and funding acquisition. W.D.: conceptualization and funding acquisition. Z.-G.H.: funding acquisition and supervision.

## Declaration of interests

The authors declare that this study was conducted in the absence of any commercial or financial relationships that could be construed as potential conflicts of interest.

## STAR★Methods

### Key resources table

**Table:** 

REAGENT or RESOURCE	SOURCE	IDENTIFIER
**Biological samples**		

Human blood samples	This paper	N/A

**Critical commercial assays**		

Urine luteinizing hormone (LH) detection kit	DAVID	Cat# 20162400112

**Deposited data**		

fMRI data	This paper	N/A

**Software and algorithms**		

MATLAB	MathWorks	https://www.mathworks.com/
SPM12	Wellcome Center for Human Neuroimaging	https://www.fil.ion.ucl.ac.uk/spm/software/download/
GRETNA V2.0	The Brainnetome Lab, National Key Laboratory of Cognitive Neuroscience and Learning, Beijing Normal University	https://www.nitrc.org/projects/gretna/
G∗Power 3.1.9.7	Heinrich Heine University Düsseldorf	https://www.psychologie.hhu.de/arbeitsgruppen/allgemeine-psychologie-und-arbeitspsychologie/gpower
Code	This paper	Zenodo:https://doi.org/10.5281/zenodo.20278381

**Other**		

3.0 T MRI scanner (GE SIGNA HDxt)	GE Healthcare	N/A

### Experimental model and study participant details

#### Human participants

Before the experiment, all participants were provided with information regarding the study procedure and were required to provide written informed consent. The Ethical Review Committee of the First Affiliated Hospital of Xi’an Jiaotong University approved this study, which was conducted in accordance with the principles of the Declaration of Helsinki (approval no.2017-154). All participants were Chinese Asian and biologically female. Biological sex was determined based on self-report and clinical screening information related to menstrual history. Because PDM is a female-specific gynecological pain condition associated with the menstrual cycle, the study population was restricted to female participants. Accordingly, the independent effects of sex/gender could not be separately evaluated. Potential participants were screened and diagnosed by a gynecologist at the Department of Obstetrics and Gynecology. The inclusion criteria for PDM group were as follows: (1) patients fulfilled the diagnostic criteria of the American College of Obstetricians and Gynecologists (lower abdominal pain during menstruation that interfered with daily activities, but without any underlying pathological abnormality within or outside of the uterus), (2) right-handed women aged 18–30 years with regular menstrual cycles (27–32 days). (3) Patients rated an average cramping pain level of ≥4 in the past 6 months. The visual analog scale (VAS)^75^ (0 = no pain; 10 = worst imaginable pain) was used to assess the average intensity of pelvic pain during menstruation over the previous 6 months.

The exclusion criteria were (1) the presence of structural abnormalities in the clinical MRI images of the brain and pelvis, (2) the use of medications, including hormonal supplements, Chinese medicine, or any other drugs that affect the central nervous system, within 6 months before MRI, (3) a history of chronic illness, neurological disease, psychiatric disorders, or left-handedness, (4) the presence of a metal or pacemaker implant, (5) pregnancy or immediate plans for pregnancy, (6) alcohol or drug abuse, and (7) claustrophobia. A total of 46 patients from local colleges and universities, met the inclusion criteria and completed three times MRI scans. The MRI scans were performed during the menstrual phase (day 1–2), periovulatory phase (day 13–15), and the luteal phase (day 19–20). Neither analgesics nor antidepressant was taken 24 h before the MRI scanning. Participants with head movements greater than 2 mm or 2° in any of the three scans were excluded. Patients were required to maintain a detailed menstrual cycle diary for at least two consecutive cycles prior to the study. Urine kits were used to measure surges in luteinizing hormone (a precursor of ovulation) levels to determine the ovulatory phase. The average pain intensity of each phase was assessed using the VAS after every MRI scan. Additionally, the Self-Rating Anxiety Scale (SAS) and the Self-Rating Depression Scale (SDS) are employed to assess patients’ anxiety and depressive state.^76^^,^^77^ Demographic and clinical characteristics of the included participants, including sex, BMI, age at menarche, menstrual cycle, and duration of menstrual pain, are presented in Table 1.

### Method details

#### Measurement of PGF2α levels

Prior to each MRI scan, nurses performed venipuncture at the antecubital vein to collect blood samples from patients. All blood samples were collected into 5-mL tubes containing ethylenediaminetetraacetic acid (EDTA). The tubes were centrifuged at 2,500 revolutions per minute at 4°C for 10 min, and the sera were stored at −70°C. These samples were subsequently analyzed by qualified laboratory technicians to measure PGF 2α levels. To reduce intra-individual variability, all samples for each participant were assayed together.

#### MRI data acquisition

A 3.0 T GE scanner (GE SIGNA HDxt, Milwaukee, WI, USA) was used to acquire brain images. The scanner was fitted with an 8-channel phased-array head coil. Participants were scanned at the Department of Medical Imaging of the First Affiliated Hospital of Xi’an Jiaotong University. Participants were instructed to keep their head immobile, relax, close their eyes, remain awake, and not to think of anything in particular. BOLD functional images were obtained using a T2∗-weighted single-shot gradient echo-planar-imaging (EPI) sequence with the following parameters: 64 × 64 matrix, repetition time (TR) = 2,000 ms, echo time (TE) = 30 ms, flip angle = 90°, FOV = 240 × 240 mm, and 30 contiguous slices 5 mm thick. In total, 185 functional volumes were obtained.

#### FMRI data preprocessing

The rs-fMRI data preprocessing was conducted on MATLAB (Mathworks, Natick, MA, USA) using SPM12 (https://www.fil.ion.ucl.ac.uk/spm/software/download/) and GRETNA V2.0 (https://www.nitrc.org/projects/gretna/).^78^ Initial processing included removal of the first five volumes to eliminate signal instability from scanner equilibration. Subsequent processing of the retained 180 volumes per subject involved: (1) slice timing correction; (2) head motion realignment; (3) affine transformation, and nonlinear registration^79^ to the standard EPI template in the Montreal Neurological Institute (MNI, Quebec, Canada) space, with 3 mm isotropic voxels.; (4) visual quality assessment of normalized images; (5) spatial smoothing with 6 mm FWHM Gaussian kernel; (6) first-order detrending.

Subsequently, independent bandpass filtering was applied to extract fMRI signals in the 0.01–0.08 Hz frequency band and its two sub-bands, including slow-5 (0.01–0.027 Hz) and slow-4 (0.027–0.073 Hz).^27^^,^^80^ Finally, nuisance regression was performed to control for the Friston 24 motion parameters,^81^ mean white matter signal, mean cerebrospinal fluid signal, and global brain signal. FMRI data exhibiting a maximum displacement exceeding 2 mm in the x, y, or z direction or an angular motion greater than 2° were excluded from the analysis.

#### Dynamic CAP analyses

CAP analysis is a data-driven method that utilizes the k-means clustering to identify recurring brain states across time points, characterized by similar whole-brain coactivation patterns within each frequency band. We utilized the Human Brainnetome Atlas (HBA), which provides a comprehensive 246 parcels of the brain. A brain volume refers to a single 3D scan captured at a time point, there were 180 volumes for each subject’s preprocessed fMRI data, and each volume was characterized by the BOLD signal intensity of 246 ROIs. The timeseries for each ROI were independently normalized using *Z* score, with the absolute value of Z indicating the deviation of activation from its baseline. The details of generating the *Z* score are provided in the supplementary materials. The CAP analysis pipeline is illustrated in Figure 1A. The BOLD signal intensities of the 246 brain regions (normalized to *Z* score) of each brain state formed a one-dimensional vector. For each menstrual-phase scan of every patient, the two-dimensional normalized BOLD signal matrix X_*i*_ (180 time points ✕ 246 ROIs) was obtained from the scan acquired during a specific menstrual phase of patient (i).^82^^,^^83^ Next, a two-dimensional matrix T was obtained, where the number of time points is 180 and N was the number of scans. The analysis was conducted by combining fMRI data from all three menstrual phases. Therefore, N represents the total number of scans obtained from all participants across the three menstrual phases.

$$T={\left[X_{1}X_{2}\;\ldots \;X_{N}\right]}^{T}$$

Subsequently, the brain states (180 ✕ N) were divided into k clusters using k-means clustering, and highly correlated vectors were grouped into a cluster. The brain states within each cluster were averaged to generate a CAP state. This process identifies the recurring brain states of coactivation that emerge across participants over time. The cluster number (k), representing the number of CAP states, was selected from 3 to 11 with a step length of 1. The silhouette coefficient method was used to identify the optimal k.^84^

Participant-level metrics of the CAPs were calculated to evaluate the dynamic properties within and between the CAP states. We assessed brain dynamic alterations in patients with PMD by calculating the statistical differences across the three phases in the metrics of fraction of time, persistence of time, counts, and transitions (Figure 1B). Fraction of time is defined as the proportion of time that each CAP state occurred throughout the entire scan. The persistence of time indicates the average number of consecutive time points during which an individual remains in each state. The counts indicate the times of the occurrence of a state throughout the scan. The transitions represented the total number of switches between CAP states across the scan.^21^^,^^24^^,^^83^^,^^85^

#### The details of generating the z-scores

The time series of each ROI was normalized by using *Z* score, and the mean of the time series represented its baseline activation level (Z = 0). As the existence of opposite CAP pairs, the absolute Z value of each ROI indicated the amplitude of deviation from the baseline, and was defined as activation deviation in this work. A larger activation deviation means a stronger positive activation or stronger negative deactivation. Specifically, positive Z values indicate activation, whereas negative Z values indicate deactivation.

In generating the z-scores for each region of interest (ROI), we first calculated the mean and standard deviation of the time series for each ROI across all time points. The *Z* score for each time point in the ROI’s time series was then computed using the formula:

$$z=\frac{\left(X-\mu \right)}{\sigma }$$

where X is the signal amplitude of an ROI at a specific time point, μ is the mean of the time series for the ROI, and σ is the standard deviation of the time series for the ROI. This method standardizes the time series data by transforming it into a distribution with a mean of zero and a standard deviation of one, thus allowing us to assess the deviation of each time point’s activation from the baseline level (where Z = 0). The absolute z-values derived from this process indicate the amplitude of activation or deactivation relative to this baseline, defining what we term “activation deviation” in our study.

### Quantification and statistical analysis

#### Sample size calculation

The required sample size was estimated using G∗Power (version 3.1.9.7 (Universität Düsseldorf, Germany). An *a priori* power analysis was performed for a one-way repeated-measures ANOVA (within-subjects factor with three measurements). Assuming a medium effect size (f = 0.25), an alpha level of 0.05, a desired power of 0.80, a correlation among repeated measures of 0.50, the analysis indicated that a sample size of 28 participants would achieve adequate statistical power.

#### Statistical analysis

The spatial similarity of each brain state was assessed using Pearson correlation between frequency bands. The repeated-measures ANOVA was performed to evaluate the effect of frequency bands on temporal dynamics of identified brain states, with the false-discovery rate (FDR) for multiple comparison correction.

One-way repeated measures ANOVA was used to compare the PGF2α levels, SAS scores, SDS scores and CAP metrics of patients with PDM across the three menstrual phases. FDR correction (*p* = 0.05) was applied to account for multiple comparisons. Statistical significance in figures is indicated as ∗*p* < 0.05 and ∗∗*p* < 0.01. We further investigated the potential associations of the CAP metrics with the pain characteristics and PGF2α levels of patients with PDM by computing Pearson correlation coefficients or Spearman’s rank correlation coefficients, depending on the normality of the data. A statistically significant threshold of *p* < 0.05 was set for all correlation analyses. Specifically, we focused on menstrual phases demonstrating statistically significant variations in the CAP metrics. Subsequently, we conducted a correlational analysis to examine the association between interphase discrepancies in the corresponding CAP metrics and differential manifestations in VAS scores alongside PGF2α levels.

## Supplemental information

Table S1. Activated and deactivated brain regions in CAP states, related to Figure 2 Brain regions are labeled according to the Brainnetome Atlas. Abbreviations of brain regions follow the atlas nomenclature (e.g., MFG, middle frontal gyrus; IFG, inferior frontal gyrus; SFG, superior frontal gyrus; IPL, inferior parietal lobule; PoG, postcentral gyrus; INS, insula; MTG, middle temporal gyrus; ITG, inferior temporal gyrus; STG, superior temporal gyrus; PrG, precentral gyrus; PCun, precuneus; CG, cingulate gyrus; SPL, superior parietal lobule; MVOcC, medial ventral occipital cortex; OrG, orbital gyrus). DMN, default mode network; SN, salience network; CEN, central executive network; DAN, dorsal attention network; SMN, sensorimotor network; VN, visual network; LN, limbic network.
